# Primary solid lung cancerous nodules with different sizes: computed tomography features and their variations

**DOI:** 10.1186/s12885-019-6274-0

**Published:** 2019-11-07

**Authors:** Zhi-gang Chu, Yan Zhang, Wang-jia Li, Qi Li, Yi-neng Zheng, Fa-jin Lv

**Affiliations:** 1 Department of Radiology, The First Affiliated Hospital of Chongqing Medical University, 1# Youyi Road, Yuanjiagang, Yuzhong district, Chongqing, China; 2 Department of Radiology, Chongqing Three Gorges Medical College, Chongqing, China

**Keywords:** Solid pulmonary nodule, Lung cancer, Computed tomography

## Abstract

**Background:**

The computed tomography (CT) features of small solid lung cancers and their changing regularity as they grow have not been well studied. The purpose of this study was to analyze the CT features of solid lung cancerous nodules (SLCNs) with different sizes and their variations.

**Methods:**

Between February 2013 and April 2018, a consecutive cohort of 224 patients (225 nodules) with confirmed primary SLCNs was enrolled. The nodules were divided into four groups based on tumor diameter (A: diameter ≤ 1.0 cm, 35 lesions; B: 1.0 cm < diameter ≤ 1.5 cm, 60 lesions; C: 1.5 cm < diameter ≤ 2.0 cm, 63 lesions; and D: 2.0 cm < diameter ≤ 3.0 cm, 67 lesions). CT features of nodules within each group were summarized and compared.

**Results:**

Most nodules in different groups were located in upper lobes (groups A − D:50.8%–73.1%) and had a gap from the pleura (groups A − D:89.6%–100%). The main CT features of smaller (diameter ≤ 1 cm) and larger (diameter > 1 cm) nodules were significantly different. As nodule diameter increased, more lesions showed a regular shape, homogeneous density, clear but coarse tumor–lung interface, lobulation, spiculation, spinous protuberance, vascular convergence, pleural retraction, bronchial truncation, and beam-shaped opacity (*p* < 0.05 for all). The presence of halo sign in all groups was similar (17.5%–22.5%; *p* > 0.05).

**Conclusions:**

The CT features vary among SLCNs with different sizes. Understanding their changing regularity is helpful for identifying smaller suspicious malignant nodules and early determining their nature in follow-up.

## Background

Lung cancer is the most common type of tumor and the leading cause of cancer-related mortality worldwide [1, 2]. Pulmonary nodules are one of the main presentations of lung cancer; therefore, they have always been the key point of research. Based on their density features on computed tomography (CT) images, cancerous nodules can be generally divided into solid and subsolid nodules, each with significantly different morphological and pathological features [3]. Several studies have been conducted on the differential diagnosis of benign and malignant subsolid nodules [4–8] and the solid ones [9–13]. Compared with subsolid lung cancerous nodules, the solid ones have a worse prognosis because of their rapid growth [14–16] and earlier metastases [16–19]. Therefore, early identification of malignant solid nodules, particularly the smaller ones, based on CT features, is of great value for their prognosis.

Among the various CT features, spiculation, lobulation, vascular convergence, and pleural retraction have been associated with malignancy in lung cancer [6, 20–25]; therefore, they are helpful in differentiating benign from malignant nodules. However, these features are usually absent in smaller nodules, making their diagnosis challenging. Management of smaller nodules has mainly relied on clinical follow-up, using changes in nodule size to determine benignity or malignancy [26–28]. However, there is no systematic report on changes other than those in size, including shape, density, margins, interface, and internal characteristics; furthermore, the clinical significance of these changes remains unclear. Therefore, further investigating CT features associated with the changing regularity of solid lung cancerous nodules (SLCNs) with different sizes has the potential to greatly improve early detection of malignant nodules.

To date, no study has reported imaging features associated with the development of primary SLCNs. Therefore, the purpose of this study was to investigate and summarize CT manifestations of primary SLCNs and their differences based on the nodules size and to provide a reference for early and accurate identification of potentially malignant small nodules.

## Methods

### Patients

The present study enrolled patients with pathologically confirmed peripheral lung cancer between February 2013 and April 2018. All patients underwent chest CT examination before surgery. Inclusion criteria were met by patients with (1) lesions comprising nodules with diameter ≤ 3 cm; (2) an interval of 1 month between chest CT scan and surgery, and (3) lesions not treated with antitumor therapy before CT examination. Patients were excluded if their CT images were of poor quality (5 cases) or if the lesions were metastatic lung cancers (14 cases). A total of 224 patients (225 lesions) were included in the study.

### CT examinations

All patients were examined using a 64-slice spiral CT scanner (SOMATOM Definition Flash, Siemens, Germany) with the following settings: tube voltage, 140kVp; tube current calculated according to individuals’ weight, height, and body mass index; rotation time, 0.5 s; pitch, 1.0; collimation, 0.6 mm; slice thickness and interval for axial images, 5 mm and 5 mm, respectively; and reorganization interval, 1 mm. Upon CT examination, patients were put in the supine position with both hands near the head. Image acquisition was performed from the level of the thoracic inlet to inferior to the costophrenic angle. Images were obtained with mediastinal window (width, 400 HU; level, 30 HU) and lung window (width, 1500 HU; level, − 600 HU) settings.

### Image analysis

All patients’ CT data were reviewed on a workstation (Advantage Workstation 4.6; GE Healthcare) by two senior chest radiologists who were blinded to the pathological results of lesions. Interpretation discrepancy, if any, was resolved by consensus.

The followings features of the lesions were evaluated on CT images: size (average of the maximal long-axis and perpendicular maximal short-axis dimension); distribution in different lobes (left superior and inferior lobes and right superior, middle, and inferior lobes); location (clinging to the pleura or not); shape (regular: oval, round, and polygonal or irregular); internal features (calcification, air bronchogram, vacuole sign, or cavity); density (homogenous or heterogeneous); margins (lobulation, spiculation, spinous protuberance); and tumor–lung interface (coarse, unclear, or smooth). In addition, peripheral lesion areas were also evaluated, including vascular convergence, pleural retraction, bronchial truncation, and beam–shaped opacity. Pleural effusions and lymph nodes in the hilum and mediastinum were further evaluated. Enlarged mediastinal and hilar lymph nodes were generally defined as those with diameter > 1 cm in short axis on chest CT scans.

For investigating the differences in CT features of SLCNs with different sizes, especially for the smaller ones, nodules were divided into four groups based on tumor size: Group A: diameter ≤ 1.0 cm; Group B: 1.0 cm < diameter ≤ 1.5 cm; Group C: 1.5 cm < diameter ≤ 2.0 cm; and Group D: 2.0 cm < diameter ≤ 3.0 cm.

### Statistical analysis

Clinical data and CT features of nodules were statistically analyzed for each group. Continuous variables were expressed as mean ± standard deviation, whereas categorical variables were expressed as absolute number and percentage. Independent sample *t*-tests were used to compare age among different groups. Chi-square test was used to compare the differences in patients’ clinical and pathological data, distribution and location of nodules, and various CT features among the groups. Tukey–Kramer test was used for multiple comparisons among the groups. A *p*-value less than 0.05 was considered statistically significant. All statistical analyses were performed using the statistical software SPSS (version 22.0 for Windows, SPSS Inc., Chicago, Illinois, USA).

## Results

### Patient characteristics

Among the 224 patients, 129 (57.6%) were male and 95 (42.4%) were female. Patients were aged 38–83 years, with an average age of 61.7 ± 9.5 years. A total of 127 (56.7%) cases had cough, expectoration, hemoptysis, chest pain, or chest tightness. The number of SLCNs in groups A, B, C, and D was 35, 60, 63, and 67, respectively. Table 1 summarizes the clinical and pathological data of patients included in each group. No significant differences were found in gender, age, clinical symptoms, smoking history, and histopathological types of lung cancer among the four groups (*p* > 0.05).

**Table 1:** Patients’ clinical and pathological data

	Group A (*n* = 35)	Group B (*n* = 60)	Group C (*n* = 63)	Group D (*n* = 67)	*p-value*
Age (years)	59.3 ± 9.2	63.0 ± 10.6	60.9 ± 8.3	64.0 ± 9.0	0.841
Male	18 (51.4)	35 (58.3)	36 (57.1)	41 (61.2)	0.821
Smokers	12 (34.3)	20 (33.3)	29 (46.0)	34 (50.8)	0.157
Clinical symptoms	16 (45.7)	32 (53.3)	42 (66.7)	37 (55.2)	0.204
Pathological types					
Adenocarcinoma	32 (91.4)	55 (91.7)	51 (81.0)	56 (83.6)	0.245
Squamous cell carcinoma	1 (2.9)	5 (14.3)	10 (15.9)	6 (9.0)	0.191
Others	2 (5.7)	0 (0)	2 (3.2)	5 (7.5)	0.174

The data are expressed as n (%)

### Lesion distribution and location

Nodule distribution and location are summarized in Table 2. In all four groups, nodules were mainly distributed in the upper lobes (50.8–73.1%) and most of them did not cling to the pleura (89.6–100%).

**Table 2:** Distribution and location of nodules

	Group A (*n* = 35)	Group B (*n* = 60)	Group C (*n* = 63)	Group D (*n* = 67)	*p-value*
Right lung	28 (80.0)	33 (55.0)	31 (49.2)	42 (62.7)	0.022
SL	15 (42.9)	20 (33.3)	16 (25.4)	29 (43.3)	0.137
ML	5 (14.3)	2 (3.3)	4 (6.4)	5 (7.5)	0.252
IL	8 (22.9)	11 (18.3)	11 (17.5)	8 (11.9)	0.540
Left lung	7 (20)	27 (45)	32 (50.8)	25 (37.4)	0.022
SL	6 (17.1)	17 (28.3)	16 (25.4)	20 (29.9)	0.550
IL	1 (2.9)	10 (16.7)	16 (25.4)	5 (7.5)	0.005
SLs of both lungs	21 (60.0)	37 (61.7)	32 (50.8)	49 (73.1)	0.074
ILs of both lungs	9 (25.8)	21 (35)	27 (42.9)	13 (19.4)	0.026
Relationship with pleura					
Not clinging to pleura	35 (100.0)	58 (96.7)	58 (92.1)	60 (90)	0.114
Clinging to pleura	0 (−)	2 (3.33)	3 (4.76)	6 (8.96)	0.114

*SL* Superior lobe, *ML* Middle lobe, *IL* Inferior lobe

The data are expressed as n (%)

### CT characteristics of lesions and their surroundings

**Fig. 1:**
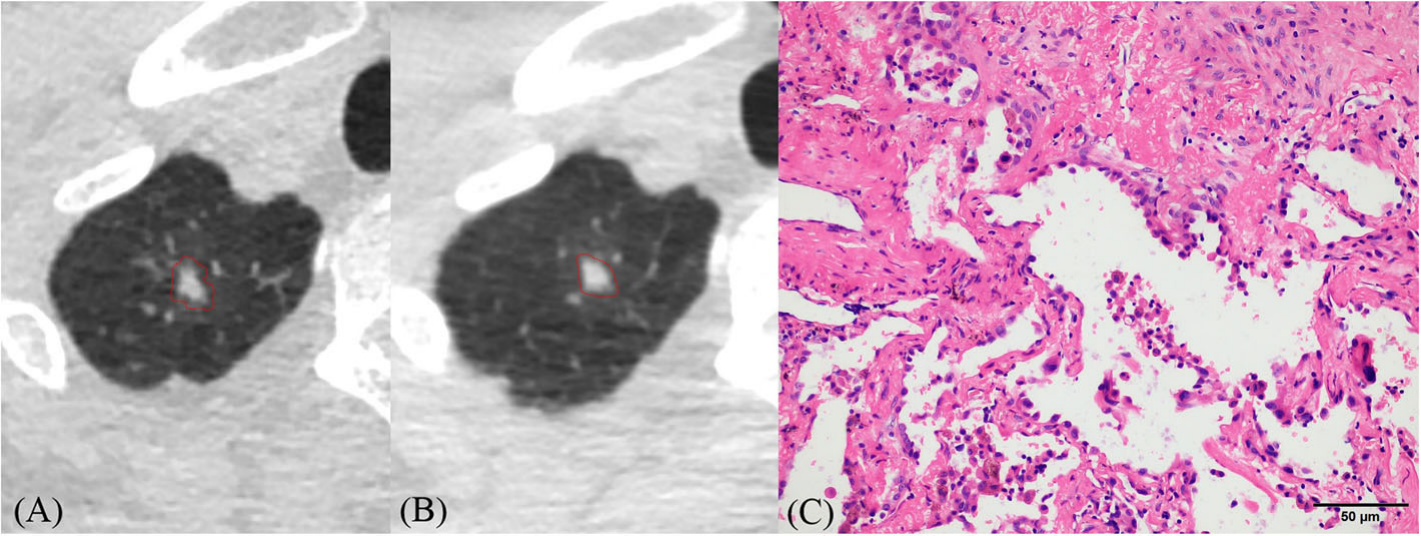
**a**–**c** CT images of lung adenocarcinoma. **a** CT image shows an irregular solid nodule (6 × 4 mm) with blurred margin located in the apical segment of the right upper lobe. **b** One year later, the size of this nodule increased (8 × 6 mm) and its shape and margin became more regular (triangle) and clearer than before. **c** Histopathologic analysis of the resected nodule revealed microinvasive adenocarcinoma

**Fig. 2:**
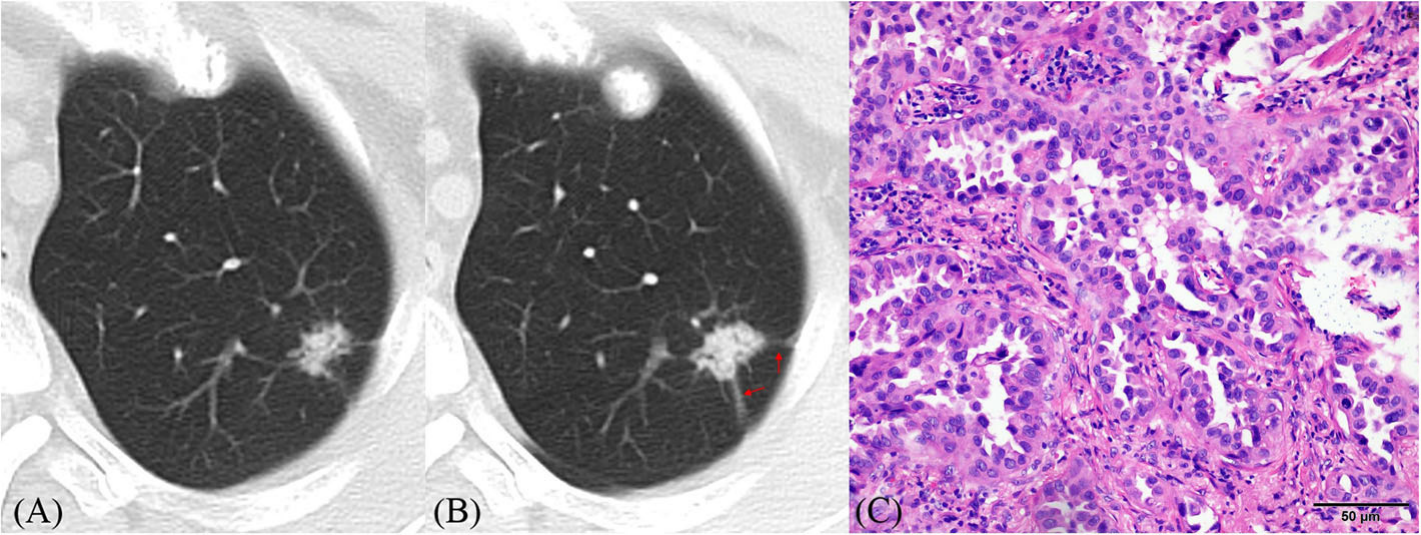
**a**–**c** CT images of lung adenocarcinoma. **a** CT image shows an irregular solid nodule (10 × 15 mm) with heterogeneous density located in the apico-posterior segment of the left upper lobe. **b** One year later, its size (14 × 16 mm) and density increased; the tumor–lung interface was clearer and spiculation (red arrows) was more obvious than before. **c** Histopathologic analysis of the resected nodule revealed invasive adenocarcinoma

**Fig. 3:**
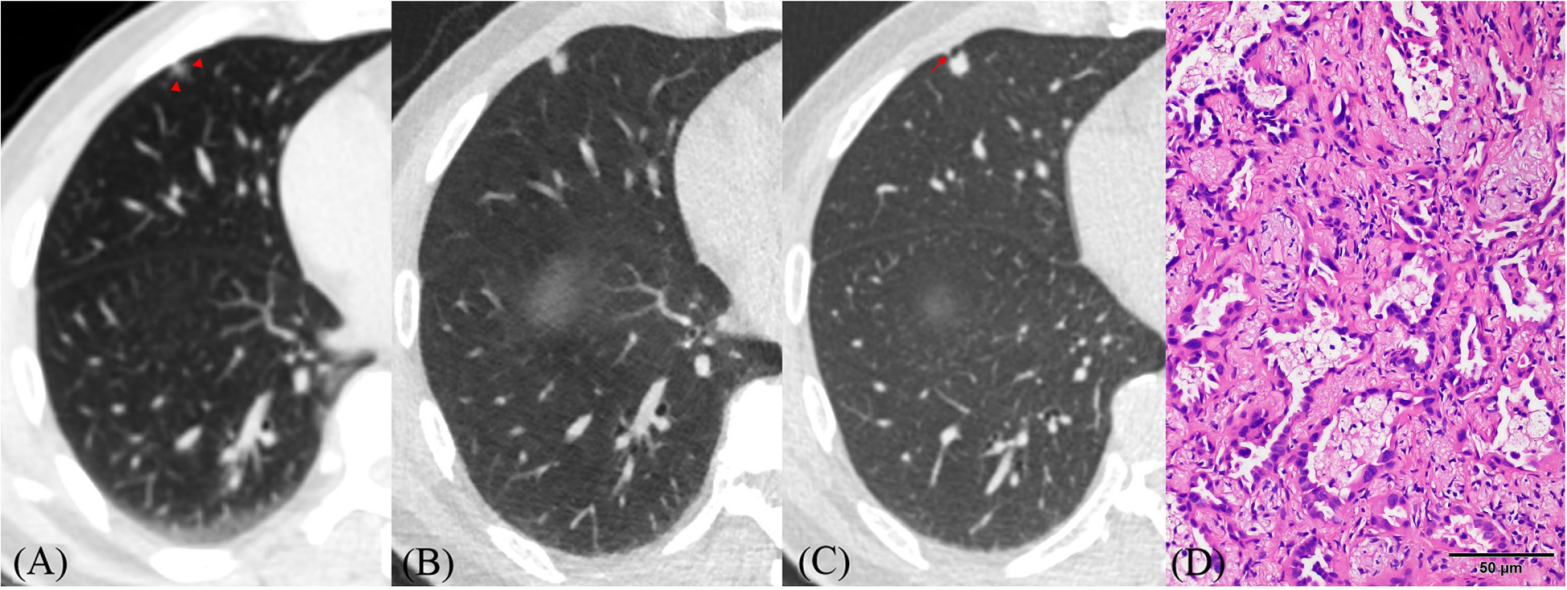
**a**–**d** CT images of lung adenocarcinoma. **a** CT image shows a nodule (7.0 × 5.0 mm) with heterogeneous density and blurred tumor–lung interface (red arrowheads) located in the subpleural zone of the right middle lobe. **b** One year and a half later, it grew a little (8.0 × 6.0 mm) but its density significantly increased. **c** Two years and a half later, its size (8.0 × 8.0 mm) slightly increased but margins became clearer than before. Lobulation and pleural indentation (red arrow) are obvious. **d** Histopathologic analysis of the resected nodule revealed adenocarcinoma without significant invasion

**Fig. 4:**
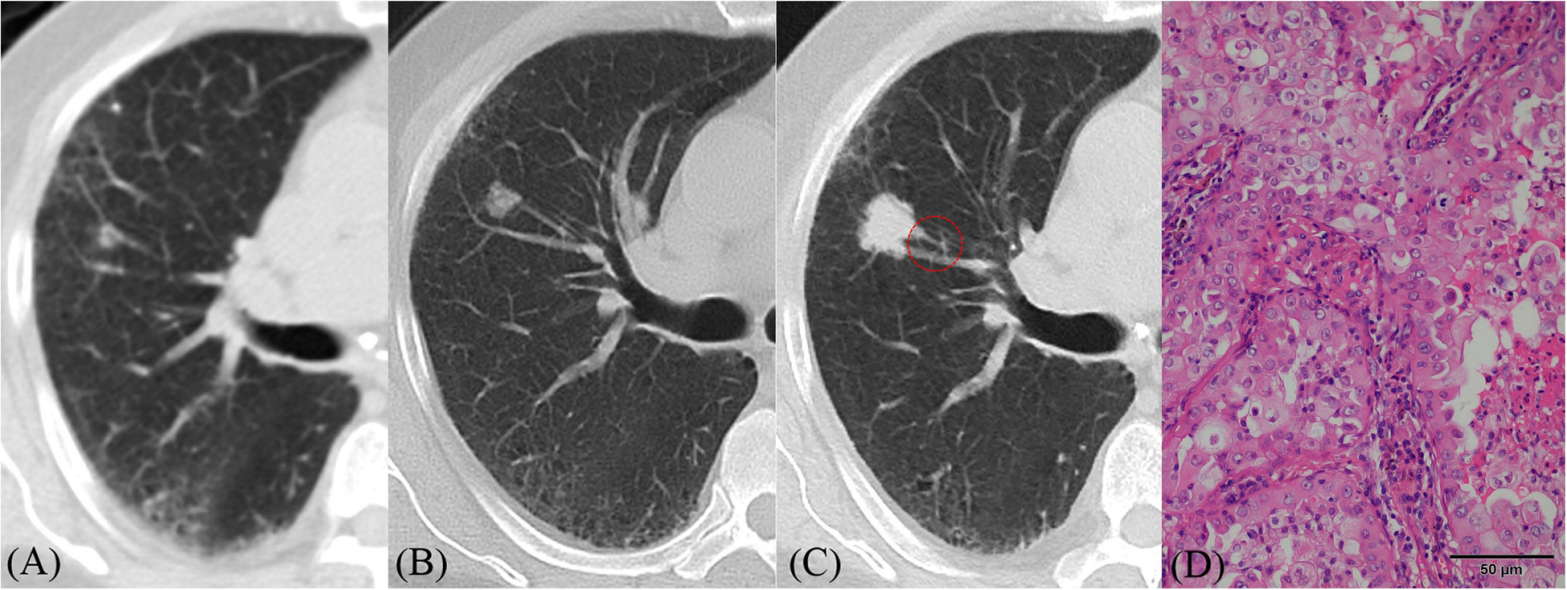
**a**–**d** CT images of lung adenocarcinoma. **a** CT image shows a small nodule (5 mm) beside a blood vessel in the right upper lobe. **b** Eight months later, its size increased (10 × 11 mm) but its density was heterogeneous. **c** Fifteen months later, its size significantly increased (18 × 19 mm); furthermore, its density increased, and it became more homogeneous. Additionally, lobulation sign and peripheral vascular convergence (red circle) were positive. **d** Histopathologic analysis of the resected nodule revealed invasive adenocarcinoma

Nodule CT features are summarized in Table 3. As nodule diameter increased, more lesions showed a regular shape, homogeneous density, clear but coarse tumor–lung interface, lobulation, spiculation, spinous protuberance, vascular convergence, pleural retraction, bronchial truncation, and beam-shaped opacity (*p* < 0.05 for all) (Figs. 1, 2, 3 and 4). However, the significant differences in various CT features were mainly detected between nodules in group A (diameter ≤ 1 cm) and those in groups B, C and D (diameter > 1 cm). Additionally, the presence of halo sign in groups A and B was slightly higher than that in groups C and D, although not significantly different (*p* > 0.05). There was also no significant difference in the incidence of calcification, vacuole, and cavity among the four groups.

**Table 3:** CT features of nodules in different size

CT features	Group A (*n* = 35)	Group B (*n* = 60)	Group C (*n* = 63)	Group D (*n* = 67)	*p-value*	Sig.
Shape						
Round	9 (25.7)	14 (23.3)	15 (23.8)	15 (22.4)	0.986	–
Oval	16 (45.7)	41 (68.3)	42 (66.7)	48 (71.6)	0.060	–
Polyonal	5 (14.3)	3 (5)	4 (6.3)	2 (3.0)	0.154	–
Irregular	5 (14.3)	2 (3.3)	2 (3.2)	2 (3.0)	0.049	A/BCD
Size (mm)	8.2 ± 1.4	12.8 ± 1.3	17.7 ± 1.4	24.1 ± 2.7	/	
Heterogeneous density	18 (51.4)	15 (25)	8 (12.7)	3 (4.5)	0.000	A/BCD
Internal feature						
Calcification	0 (−)	0 (−)	1 (1.6)	1 (1.5)	0.687	–
Vacuole	5 (14.3)	5 (14.3)	4 (6.4)	11 (16.4)	0.242	–
Air bronchogram	1 (2.9)	1 (1.7)	5 (7.9)	12 (17.9)	0.005	A/BCD
Cavity	0	1 (1.7)	7 (11.1)	4 (6.0)	0.051	–
Margin						
Lobulation	10 (28.6)	22 (36.7)	36 (57.1)	47 (70.1)	0.000	AB/CD
Spiculation or spinous protuberance	7 (20)	27 (45)	36 (57.1)	47 (70.1)	0.000	A/BCD
Halo sign	7 (20)	15 (25)	11 (17.5)	12 (17.9)	0.712	–
Tumor-lung interface						
Coarse	26 (74.3)	48 (80)	55 (87.3)	64 (95.5)	0.013	A/D
Unclear	6 (17.1)	7 (11.7)	3 (4.8)	1 (1.5)	0.016	A/D
Smooth	3 (8.6)	5 (14.3)	5 (7.9)	2 (3.0)	0.553	–
Bronchial truncation	0 (−)	8 (13.3)	10 (15.9)	18 (26.9)	0.005	A/BCD
Vascular convergence	6 (17.1)	32 (53.3)	43 (68.3)	50 (74.6)	0.000	A/BCD
Beam-shaped opacity	3 (8.6)	15 (25)	15 (23.8)	26 (38.8)	0.010	A/BCD
Pleural retraction	3 (8.6)	22 (36.7)	28 (44.4)	34 (50.8)	0.000	A/BCD
Obstructive pneumonia	0 (−)	1 (1.67)	4 (6.4)	5 (7.5)	/	/

The data are expressed as n (%). Heterogeneous density indicates the density of nodules in addition to calcification, vacuole, air bronchogram, and cavity. A/BCD indicates there is significant difference between group A and B but no significant difference among groups of B, C and D. AB/CD indicates there is significant difference between group B and C but no significant difference between group A and B or group C and D. A/D indicates there is significant difference only between group A and D

### CT characteristics of suspicious malignant nodules in group a

Along with lesion distribution, location, and morphological features, the main CT characteristics indicating suspicious malignant nodules in group A are summarized in Table 4.

**Table 4:** CT features indicating suspicious malignancy for group A nodules

CT features	Numbers	Percentage (%)
SLs of both lungs^a^	21	60.0
Not clinging to pleura^a^	35	100.0
Irregular shape^b^	5	14.3
Heterogeneous density^b^	18	51.4
Lobulation^c^	10	28.6
Spiculation or spinous protuberance^c^	7	20
Coarse tumor-lung interface^a^	26	74.3
Unclear tumor-lung interface^b^	6	17.1
Halo sign^a^	7	20

*SL* Superior lobe, ^a^ indicates the common features for group A-D nodules; ^b^ indicates these features are relatively common in group A nodules. ^c^ indicates these features are relatively rare in group A nodules

### Lymph nodes in the hilum and mediastinum and pleural effusion

As nodule size increased, the incidence of enlarged lymph nodes in the hilum or mediastinum also gradually increased (8.6, 18.3, 27.0, and 46.3% for groups A, B, C, and D, respectively; *p* < 0.001). The incidence of pleural effusion was extremely low in all four groups.

## Discussion

This study showed a similar distribution of SLCNs in the different groups categorized based on nodule size. Lung cancers are generally believed to be mainly distributed in the upper lobes [29–31]. Regarding nodule location, most nodules, particularly the smaller ones, were not found to be connected with the adjacent pleura. It appeared that the space between the tumor and pleura subsists until the nodules grow large enough to occupy it [32]. It is possible that a SLCN is typically derived from the distal bronchus, and a gap exists between the lesion and the pleura. These findings show that smaller nodules clinging to the pleura, which are usually detected on chest CT scan, are less likely to be lung cancer.

In this study, the CT features of smaller lesions (≤ 1 cm) were usually different from those of the bigger ones (> 1 cm). As nodule size increased, the lesions acquired a more regular shape and the margins and surrounding features also gradually increased. It may be because a nodule becomes more regular with an increase in size, and the limitation of the surrounding structure becomes more obvious. Lobulation, spiculation, pleural retraction, and vascular convergence are considered common signs of malignancy in lung cancer [6, 20–25]. However, for smaller nodules, traction and invasion of surrounding blood vessels and tissues, as well as tumor and peritumoral fibrosis were not obvious. As nodules size increased, the invasion of the surrounding tissue also increased, and more surrounding signs were evident.

Siegelman et al. [33] reported that the incidence of coarse tumor–lung interface was significantly higher in lung cancers than in benign lesions. In this study, tumor–lung interface in each group was mainly coarse. Additionally, the incidence of coarse interface increased with the increasing size of the nodule. This may be because tumor cells locally infiltrate the peripheral tissue, particularly the bigger nodules. However, the smaller the nodules, the higher the incidence of unclear interface, which may be related to the relatively sparse tumor cells in the peripheral areas of those nodules. Therefore, for smaller solid nodules with unclear interface, the possibility of lung cancer cannot be completely excluded and follow-up is recommended in such cases to avoid an erroneous diagnosis.

The growth of solid lung cancer is a gradual process. The tumor cells gradually accumulate, and the lesion size continuously increases. Theoretically, tumor density is more homogeneous on plain CT scan with an increase in the lesion size. In the present study, small nodules, particularly those with diameter < 1 cm, had a higher incidence of heterogeneous density. However, the incidence of internal calcification, vacuole sign, or cavity was low in each group, as previously reported [34, 35]. Therefore, small solid nodules with heterogeneous density can be selectively observed, and lung cancer should be highly suspected once their density increases and becomes homogeneous.

Beam-shaped opacity is banded ground glass opacity, which is located on the side of the tumor close to the pleura in different directions. It is common in adenocarcinoma and highly significant in the diagnosis of lung cancer [36]. This sign may be related to the traction of the surrounding lung tissue. In this study, the incidence of beam-shaped opacity was higher in the group D, but it was significantly lower in the group A. This implicates that small nodules do not cause significant changes in surrounding structures.

Halo sign is a nonspecific sign around solid pulmonary nodules, and its border is usually clear in lung cancer lesions [37]. In this study, a well-defined halo sign was mainly located on one side of the nodule, and its incidence was slightly higher in smaller lesions. Therefore, small solid nodules not presenting with other features but well-defined halo sign should be suspected for lung cancer.

Overall, regarding the small pulmonary nodules, follow-up seems to be an effective way to discriminate their nature based on changes in CT features. A recent study has confirmed that quantitative image features (“radiomics”) can also help discriminate benign from malignant pulmonary nodules [38]. Additionally, quantitative radiomic signatures have shown the potential to reveal and predict the tumor growth rate, and they can help identify the indolent from aggressive lung cancer [39]. Thus, radiomics may provide a new way for evaluating and managing indeterminate pulmonary nodules in the future.

This study had several limitations. The evaluation of changing regularity of CT features for SLCNs was performed by comparing grouped nodules with different sizes rather than following only one group of lesions. Thus, results obtained here should be confirmed in clinical practice. Additionally, the pathological types of SLCNs varied but showed no significant differences among different groups. Therefore, present results seem to represent a general, rather than a specific tumor type. It should be noted that some tumor types within smaller samples may not conform to the general morphological development.

## Conclusion

Morphological changes in SLCNs exhibited some degree of regularity in their growth process. With diameter increasing, more nodules showed a regular shape, homogeneous density, clear but coarse tumor–lung interface, and margin or peripheral signs. Thus, larger nodules could be easily diagnosed due to the presence of more features. However, malignancy should be suspected for smaller nodules located in the upper lobe that are not clinging to the pleura and are showing irregular shape, heterogeneous density, unclear tumor–lung interface, or a well-defined halo sign. Understanding the changing regularity of SLCNs is helpful for identifying smaller suspicious malignant nodules and for early determination of their nature during follow-up.

## Data Availability

Please contact author (Zhi-gang Chu) for data requests.

## Abbreviations

CT: computed tomography
SLCN: solid lung cancerous nodule
